# T cell receptors specific for an imatinib-induced mutation in BCR-ABL for adoptive T cell therapy

**DOI:** 10.3389/fimmu.2025.1518691

**Published:** 2025-01-27

**Authors:** Meng-Tung Hsu, Gerald Willimsky, Leo Hansmann, Thomas Blankenstein

**Affiliations:** ^1^ Molecular Immunology and Gene Therapy, Max-Delbrück-Center for Molecular Medicine in the Helmholtz Association, Berlin, Germany; ^2^ Charité-Universitätsmedizin Berlin, Corporate Member of Freie Universität Berlin, Humboldt-Universität zu Berlin, and Berlin Institute of Health, Berlin, Germany; ^3^ German Cancer Research Center, Heidelberg, Germany; ^4^ German Cancer Consortium, partner site Berlin, Berlin, Germany; ^5^ Department of Internal Medicine III, University Hospital Regensburg, Regensburg, Germany

**Keywords:** chronic myeloid leukemia, imatinib, resistance, neoantigen, T cell receptor

## Abstract

BCR-ABL kinase is the major oncogenic driver of chronic myeloid leukemia (CML). Tyrosine kinase inhibitors (TKIs), which are highly potent in targeting BCR-ABL, are currently used as first-line treatment. Although TKIs are effective, drug resistance caused by the emergence of drug-selected secondary mutations in BCR-ABL remains a major problem for relapse, especially in patients with compound mutations. In this study, we aimed to investigate potential neoepitopes derived from mutated BCR-ABL and to generate neoepitope-specific TCRs for adoptive T cell therapy. Two candidate peptides derived from the E255V and the T315I mutation (designated ABL-E255V and ABL-T315I) were selected for study based on their *in silico* predicted binding affinity to HLA-A2. By immunizing transgenic mice that express a diverse human T cell receptor (TCR) repertoire restricted to HLA-A2, we detected CD8+ T cell responses against the ABL-E255V, but not the ABL-T315I peptide. From immune responding mice, two E255V-specific TCRs were isolated. Human CD8+ T cells were engineered to express the specific TCRs for characterization, in which one TCR was identified as a therapeutic candidate due to its superior avidity and lack of detectable off-target reactivity. Importantly, we demonstrated that the ABL-E255V neoepitope was naturally processed and presented. In summary, our results demonstrate that HLA-A2^+^ CML cells harboring the E255V mutation can be targeted by specific TCRs, which may benefit patients who are highly resistant to available TKIs due to compound mutations.

## Introduction

The chimeric *BCR-ABL* gene, which results from a reciprocal chromosomal translocation t(9;22)(q22;q11), is a hallmark of chronic myeloid leukemia (CML) (1, 2). As an oncogenic driver, the encoded BCR-ABL exhibits constitutive tyrosine kinase activity, which leads to several oncogenic phenotypes and disease progression (3, 4).

Tyrosine kinase inhibitors (TKIs) are currently the first-line treatment for CML. TKIs are small molecules that can effectively suppress the BCR-ABL activity by blocking its downstream activation. Over the past decades, TKIs have significantly improved long-term survival rates, however, relapse remains a problem (5, 6). Several mechanisms leading to drug resistance have been described, the most common of which is the occurrence of point mutations in the *BCR-ABL* gene (7–9). Of the reported mutations, nearly 60% are predominately located in the ABL domain, interfering with the binding of TKIs to the BCR-ABL kinase (10). Second-generation TKIs can overcome most of the mutations that cause resistance to imatinib (a first-generation TKI) (8, 9). Patients who are highly resistant to most TKIs can be treated with a third-generation TKI called ponatinib (11). Nevertheless, 50% of mutations in patients receiving sequential TKI treatment are compound mutations (12). A preclinical study has shown that the efficacy of ponatinib decreased when confronted with certain compound mutations (13), and an *in vitro* study has demonstrated that compound mutations confer varying resistance to all available TKIs, including ponatinib (14).

Since there are no effective TKIs for patients carrying compound mutations, the development of novel therapeutic strategies is necessary. Generally, allogeneic stem cell transplantation is recommended for patients who fail sequential TKI treatment (15). However, limited matched donors and age restrictions can be barriers (8, 16). Therefore, adoptive T cell therapy, which treats cancer with T cell receptor (TCR)-engineered T cells, offers another option for such patients. In CML, drug-selected mutations, also called secondary mutations, can vary between cases. Nevertheless, certain mutations are often observed in relapsed patients, such as G250A/E, Q252H, Y253F/H, E255K/V, T315I, F317L and M351T (17, 18). As these mutations are commonly shared between patients and are exclusively expressed in CML leukemic cells, they may serve as potential targets for adoptive T cell therapy.

In this study, we used AB*ab*-A2 (previous ABabDII) transgenic mice to determine T cell responses towards potential neoepitopes derived from mutated BCR-ABL and to isolate neoepitope-specific TCRs for clinical use. AB*ab*-A2 mice carry the human *TCR α* and *β* gene loci and the HLA-A2/H2-D^b^ chimeric molecule linked to human β2-microglobulin and have been validated to express a diverse and functional human TCR repertoire restricted to HLA-A*0201 (HLA-A2) (19, 20). By immunizing AB*ab*-A2 mice with selected potential neoepitopes, we successfully isolated specific TCRs against the neoepitope, ABL-E255V, and identified a therapeutic TCR candidate, T9141-TCR, based on its high sensitivity and no detectable off-target toxicity. Importantly, the recognition mediated by T9141-TCR to HLA-A2^+^/E255V^+^ cancer cells was verified. These findings suggest that the T9141-TCR can specifically target CML leukemic cells that highly express the ABL-E255V-neoepitope, thus benefiting patients who are resistant to available TKIs due to E255V-involved compound mutations.

## Materials and methods

### Cell lines

The cell lines used, including 624.Mel 38, SK.Mel 37, LB373.Mel, MZ2.Mel 43, K562 and BV173, and their maintenance are listed in the **Supplementary Table S1**. K562-A2 cell line was generated by transducing K562 cells with *HLA-A2* cDNA. The ABL-minigene-E255V-expressing variants and the SPTG4-expressing variants were generated by transducing cancer cells with ABL-minigene-E255V or *STPG4* cDNA linked to *GFP* or *mCherry* by an internal ribosomal entry site (IRES), where ABL-minigene-E255V cassette was constructed of a DNA fragment of a size of 1045 bp (position 19-1063 of ABL open reading frame, ORF) comprising the E255V mutation. The mutated CML cell clones harboring the E255V^+^ *BCR-ABL* gene were generated by CRISPR-Cas9 system. All cell culture reagents were purchased from Life Technologies unless otherwise indicated.

### Immunization of AB*ab*-A2 mice

For priming, AB*ab*-A2 mice were injected subcutaneously with 100 nmol of peptide, ABL-E255V (KLGGGQYG **V**, Genscript) or ABL-T315I (YII **I** EFMTYG, Genscript), in a 200 μl 1:1 solution of incomplete Freund’s adjuvant and PBS supplemented with 50 μg CpG. Sequential boosts were performed in 4 weeks interval. The presence of responsive CD8^+^ T cells in the peripheral blood was assessed by *in vitro* peptide stimulation and subsequent intracellular cytokine staining by BD cytofix/Cytoperm solution kit (BD biosciences) 7 days after each boost. All animal experiments were performed according to institutional and national guidelines and regulations after approval by the responsible authority (Landesamt für Gesundheit und Soziales, Berlin).

### Isolation of specific TCRs

Splenocytes from responding mice were prepared at day 10 after the last boost and cultured with 10^-9^ M peptide for 7 days. Responsive T cells were labeled by IFNγ secretion assay (Milteny) and sorted directly into RLT Plus lysis buffer (Qiagen) by flow cytometry after peptide (10^-6^ M) stimulation for 3 hr. Total RNA from sorted cells was extracted (RNeasy Plus Micro kit, Qiagen), and first-strand cDNA synthesis and 5’-RACE PCR was performed using SMARTer™ RACE cDNA amplification kit (Clontech), where TCR-specific amplification was carried out with following primers: *hTRAC* (5’-CGGCCACTTTCAGGAGGAGGATTCGGA AC-3’) or *hTRBC* (5’-CCGTAGAACTGGACTTGACAGCGGAAGTGG-3’). The RACE PCR products were cloned using a Zero Blunt TOPO PCR Cloning Kit (Life Technologies). Plasmids from individual clones were isolated and sequenced using the T3 primer (5’-AATTAACCC TCACTAAAGGG-3’) at Eurofins Genomics.

### Construction of TCR transgene cassettes

All TCR transgene cassettes were codon-optimized and synthesized by GeneArt (Life Technologies). Pairing TCR α and β chains were linked with the porcine teschovirus-1 derived self-cleavage peptide P2A, in which the human TCR constant regions were either replaced by murine constant regions or minimally murinized by introducing individual amino acids from the mouse counterparts and an additional cysteine bridge (T9141-mmc) (21–24). The transgenes were cloned into pMP71 vector using NotI and EcoRI restriction sites (25).

### TCR gene transfer by retroviral transduction

Transduction was carried out as described (26). Packaging HEK-GALV cells (HEK-293 cells expressing stably GALV-env and MLV-gag/pol) were transfected with transgene in the presence of Lipofectamine2000 (Life Technologies). Retrovirus-containing supernatant was harvested 48 and 72 hours after transfection. Human PBMCs were activated by anti-CD3 (OKT3, BD Pharmingen) and anti-CD28 (CD28.2, BD Pharmingen) antibodies in the presence of 300 U/ml hIL-2 (PeproTech) and transduced 48 and 72 hours after T cell activation by spinoculation with virus supernatant for 90 min or with virus-preloaded retronectin (Takara)-coated plates for 30 min at 800 g and 32°C. Transduced T cells were expanded for 1 week in the presence of 300 U/ml h IL-2 and rested for 3 days with 30 U/ml hIL-2 before being used for experiments.

### Genome editing with CRISPR-Cas9 system

The two CRISPR RNA (crRNA), crRNA3 (5’-AGGTCTTCACGGCCACCGTCAGG-3’) and crRNA6 (5’-CAGTACGGGGAGGTGTACGAGGG-3’), targeting the *ABL* gene were designed using CRISPOR program (27). The ribonucleoprotein (RNP) for genome editing was generated following the manufacturer’s instruction of Integrated DNA Technologies (IDT). To prepare the delivery of RNP, a total of 3×10^5^ cells were resuspended in 20 µl supplemented Nucleofector solution R (Lonza Bioscience) and mixed with 5 µl RNP, 1 µl of 100 µM single strand oligo DNA (ssODN) and 1 µl of 100 µM electroporation enhancer (IDT). The mixture was electroporated by using DN-100 program of 4D-Nucleofector device (Lonza Bioscience). The electroporated cells were immediately transferred to pre-warmed conditioned medium and cultured for expansion and selection. All CRISPR-Cas9 components were purchased from IDT. The ssODNs and the primers used for screening and sequencing are listed in **Supplementary Tables S2** and **S3** (Eurofins Genomics).

### Co-culture assay

All co-culture experiments were performed by incubating T cells and target cells for 16-18 hours at an effector-to-target (E:T) ratio of 1:1 (1×10^4^ cells of each), unless otherwise indicated. T cells cultured with 50 ng/ml Phorbol-12-myristate 13-acetate (PMA) and 5 µg/ml Ionomycin served as positive control. Human IFNγ secretion in the supernatant was measured by ELISA (BD OptEIA).

### Quantitative PCR

Total RNA was extracted using Quick-RNA™ miniprep kit (Zymo Research) and reverse transcribed using ProtoScript^®^ II First Strand cDNA synthesis Kit (BioLabs). The genes selected for Taqman^®^ gene expression assay (ThermoFisher) were BCR-ABL (b2a2, Hs03024541_ft) and ABL (exon 3-4, Hs00245445_m1). ABL (exon 8-9, Hs01104728_m1) served as an endogenous control. The qPCR was performed using QuantStudio3 Real-Time PCR system (ThermoFisher). The results were analyzed according to the comparative 2^-ΔΔCt^ Method.

### Flow cytometry

The following conjugated antibodies were used at 1:100 dilution and purchased from BioLegend: anti-hCD3 (HIT3α), anti-hCD8 (SK1), anti-hHLA-A2 (BB7.2), anti-mCD3 (145-2C11), anti-mCD8 (53.6-7), anti-mTCRβ (H57-597), anti-mIFNγ (XMG1.2). HLA-A2/ABL-E255V dextramer (Immudex) staining was performed according to the manufacturer’s instruction. Cells were analyzed by BD FACSCanto™ II (BD Biosciences) or MacsQuant (Miltenyi Biotech). Cell sorting was performed with BD FACSAria™ II (BD Biosciences). FACS data analysis was performed using FlowJo (TreeStar).

### Statistical analyses

All statistical analyses and data display were performed using GraphPad Prism version 9.4.0. The number of independent experiments, biological and technical replicates, and statistical tests used are indicated in the relevant figure legends.

## Results

### Immunizing AB*ab*-A2 mice to identify potential neoepitopes from mutated BCR-ABL

**Figure 1:**
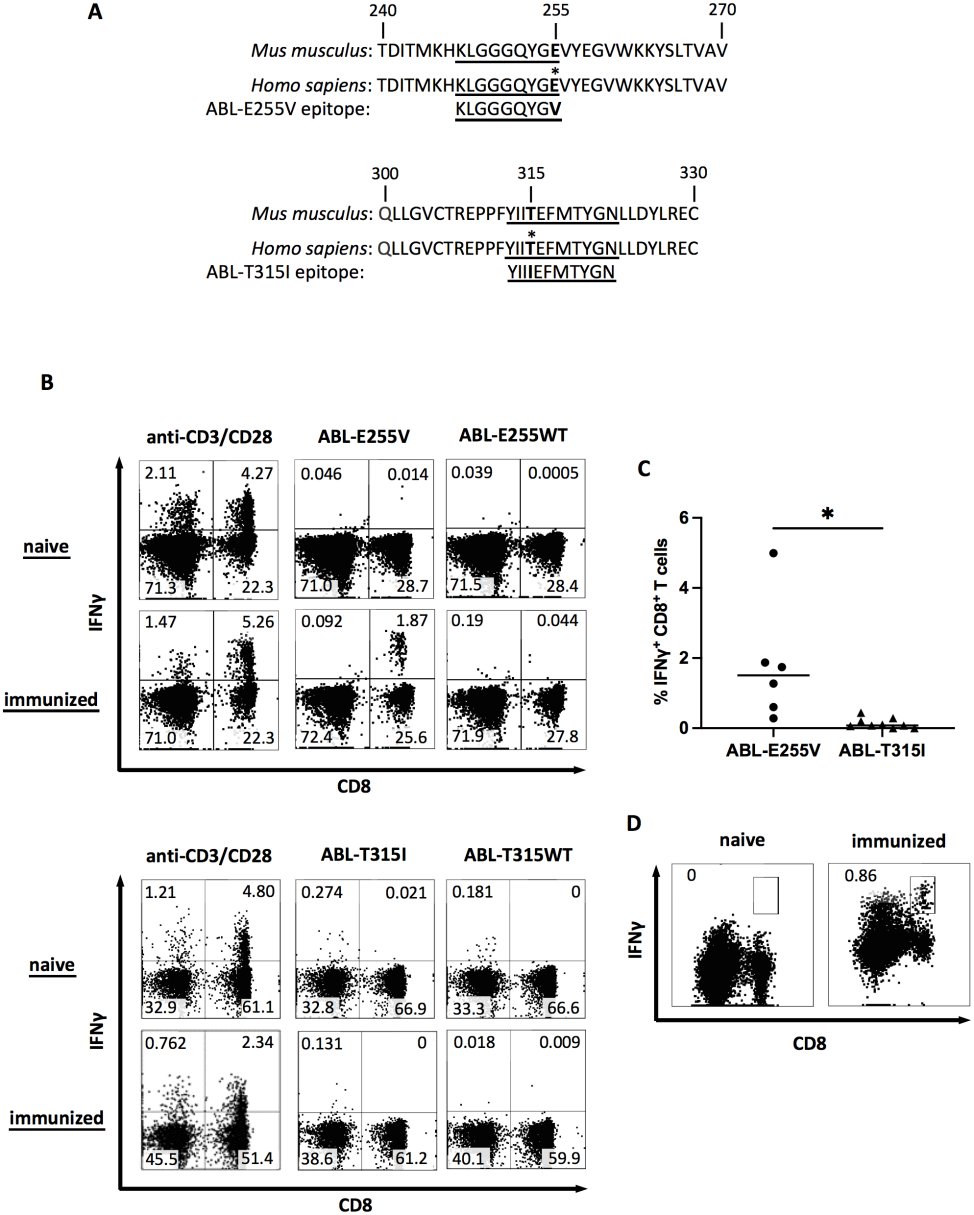
ABL-E255V but not ABL-T315I induces specific CD8^+^ T cell responses in AB*ab*-A2 mice. **(A)** The sequence of ABL-E255V and ABL-T315I, and the parental sequence in human and mouse homolog. Epitopes are underlined. **(B)** Representative plots showing intracellular IFNγ staining as indicator of mouse CD8^+^ T cell responses after peptide stimulation *in vitro* from unimmunized, ABL-E255V (n=6) or ABL-T315I (n=9) peptide immunized AB*ab*-A2 mouse. Plots were gated on CD3^+^ cells. **(C)** Summary of CD8^+^ T cell responses to immunized peptide as percentage of IFNγ-secreting CD8^+^ T cells in blood after peptide stimulation *in vitro*. Unpaired *t* test was performed for statistical analysis (*P < 0.05). **(D)** Representative plots showing clonal CD8^+^ T cell expansion detected by IFNγ secretion assay following ABL-E255V peptide stimulation *in vitro* for 10 days. Plots were gated on CD3^+^ cells.

To discover potential neoepitopes generated from mutated BCR-ABL, we evaluated frequently occurring mutations *in silico* with NetMHC 4.0 and IEDB (28–30). Two candidate peptides, ABL-E255V (KLGGGQYG **V**) and ABL-T315I (YII **I** EFMTYG), were selected for short peptide immunization (**Table 1**). The parental sequences of the two candidates were confirmed to be identical between human and mouse, ensuring that the imatinib-induced somatic mutation is the only foreign amino acid in the mice (**Figure 1A**). AB*ab*-A2 mice expressing a diverse human TCR repertoire and HLA-A2 molecules were boosted in four weeks interval. The response of each immunized mouse was tested 7 days after each boost by intracellular cytokine staining following *ex vivo* peptide re-stimulation of blood. As a result, specific CD8^+^ T cell responses were observed in several ABL-E255V-immunized mice, whereas no response was found in ABL-T315I-immunized mice (**Figures 1B, C**).

**Table 1:** Predicted binding affinity of mutated BCR-ABL peptides to HLA-A2.

Gene	Mutation	Sequence of peptide	NetMHC 4.0 IC_50_ (nM)		IEDB (SMM) IC_50_ (nM)	
			Mutant	WT	Mutant	WT
**ABL**	**E255V**	**KLGGGQYGV**	**39**	**17535**	**25**	**9206**
**ABL**	**T315I**	**YIIIEFMTYG**	**884**	**884**	**103**	**118**
ABL	E255K	KLGGGQYGK	16320	17535	3699	9206
ABL	G250A	TMKHKLGGA	14197	33932	7313	537167
ABL	G250E	KLGGEQYGE	21558	17535	104258	537167
ABL	Q252H	HYGEVYEGV	15934	16852	6473	7570
ABL	Y253H	QHGEVYEGV	21684	16852	31268	7570
ABL	Y253F	GQFGEVYEG	7807	10917	3358	4028
ABL	F317L	ELMTYGNLL	502	20983	338	12391
ABL	M351T	QISSATEYL	1110	827	1224	1033

Bold columns indicate selected candidate peptides. Underlined letters indicate mutated amino acids.

### Generation of specific TCRs from AB*ab*-A2 mice

**Figure 2:**
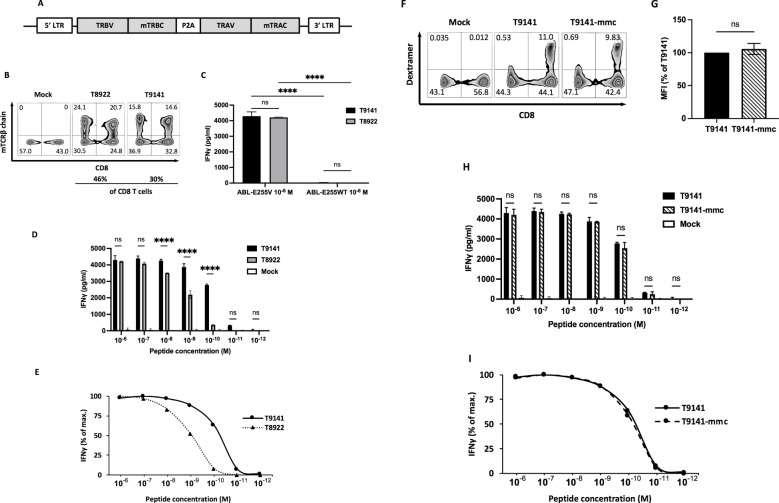
Specificity and functional avidity of ABL-E255V-specific TCRs derived from AB*ab*-A2 mice. **(A)** Schematic of constructed TCR α and β chain linked by P2A cleavage element and flanked by 5’ and 3’ long terminal repeats (LTR) in pMP71 vector. Each TCR chain contains the murine constant region instead of the human constant region. **(B)** Representative plots showing CD8 and murine TCR β constant region (mTCRβ) staining as indicators of TCR-transduced human T cells. Numbers represent percentage of cells in the respective quadrant. **(C)** IFNγ production of T9141- or T8922-transduced human T cells after co-culturing with T2 cells loaded with ABL-E255V or ABL-E255WT peptide at 10^-6^ M. Data are representative of 3 independent experiments of different donors. **(D, E)** IFNγ production of T9141- or T8922-transduced human T cells after co-culturing with T2 cells loaded with ABL-E255V at 10^-6^ to 10^-12^ M. **(D)** Data shows absolute IFNγ production. Data are representative of 3 independent experiments of different donors. **(E)** Responses of transduced human T cells were normalized to maximum IFNγ release. **(F)** Representative plots showing CD8 and peptide-HLA-A2 dextramer staining as indicators of TCR-transduced human CD8^+^ T cell. Numbers represent percentage of cells in the respective quadrant. **(G)** Mean fluorescence intensity (MFI) of peptide-HLA-A2 dextramer labeled TCR-transduced T cells. The diagram represents means of 2 donors with standard deviations (SD). Unpaired *t* test with Welch’s correction was performed for statistical analysis (ns, not significant). **(H)** IFNγ production of T9141-mmc and T9141-transduced human T cells after co-culturing with T2 cells loaded with ABL-E255V at 10^-6^ M to 10^-12^ M. Data are representative of 2 independent experiments of 2 donors. **(I)** Responses of transduced human PBMCs were normalized to maximum IFNγ release. **(C, D, H)** Mean values of duplicate cultures with SD are shown. Two-way ANOVA followed by Tukey’s multiple comparisons test was performed for statistical analysis (****P < 0.0001; ns, not significant).

Upon detection of CD8^+^ T cell responses against ABL-E255V, splenocytes from responding mice were prepared and cultured to expand specific CD8^+^ T cells. ABL-E255V-responsive CD8^+^ T cells were labelled by the mouse IFNγ secretion kit and isolated by flow cytometry (**Figure 1D**). Identified TCRα and β chains were matched and linked by P2A element for equimolar expression (**Figure 2A**). Of note, human TCR constant regions were replaced with murine constant regions, allowing preferential pairing and avoiding mispairing with endogenous TCRs (31). Finally, two TCRs, T9141 and T8922, were identified from two individual AB*ab*-A2 mice (**Table 2**).

**Table 2:** V-J segments and CDR3 regions of isolated TCRs.

TCR	V-CDR3-J	Clone frequency
T9141	TRAV38-2 - CAYRSPQGGSEKLVF - TRAJ57	100%
	TRBV2 - CASSEWPPSSYNEQFF - TRBJ2-1	83%
T8922	TRAV39*01-CAVDGDDKIIF-TRAJ30*01	46%
	TRBV7-8*01-CASSFGPVYEQYF-TRBJ2-7*01	67%

### Isolated TCRs are ABL-E255V-specific with different avidities

Human T cells were retroviral transduced to re-express the paired TCRs (**Figure 2B**). To verify the specificity of isolated TCRs, TCR-transduced T cells were co-cultured with T2 cells loaded with ABL-E255V or parental peptide ABL-E255WT (KLGGGQYG **E**), and the release of IFNγ was used as an indicator of T cell activation. As a result, both isolated TCRs specifically recognized ABL-E255V (**Figure 2C**). Next, we investigated their functionality by co-culturing TCR-transduced T cells with T2 cells loaded with titrated ABL-E255V peptide. T9141-TCR responded to ABL-E255V up to 10^-11^ M with an EC_50_ of 10^-10^ M, while T8922-TCR responded up to 10^-10^ M with an EC_50_ of 10^-9^ M (**Figures 2D, E**). For clinical purposes, T9141-TCR with superior avidity was cloned with minimally modified human constant regions (T9141-mmc-TCR) for testing (22). T9141-mmc-TCR showed comparable TCR expression by dextramer (HLA-A2/ABL-E255V) staining (**Figures 2F, G**) and achieved similar IFNγ production in response to titrated ABL-E255V peptide (**Figures 2H, I**), suggesting that the expression and functionality of the TCR were not affected by the mmc-constant region.

### T cells expressing ABL-E255V-specific TCRs recognize ABL-minigene-E255V-transduced cancer cells

**Figure 3:**
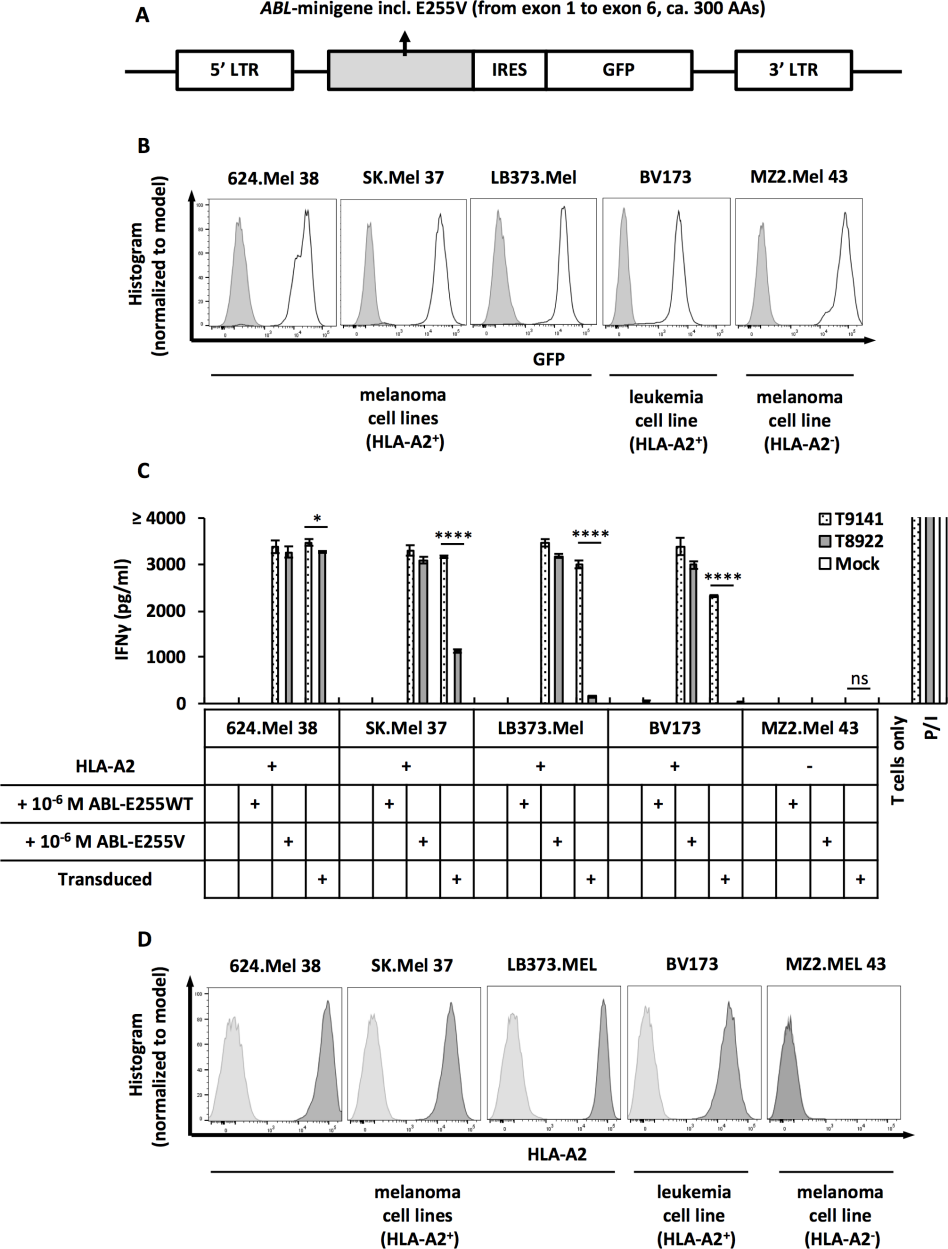
ABL-E255V epitope is endogenously processed and presented in ABL-minigene-E255V-transduced cancer cells. **(A)** Schematic of ABL-minigene-E255V linked to a GFP reporter gene by IRES and flanked by 5’ and 3’ LTR in pMP71 vector. **(B)** Expression of ABL-minigene-E255V in transduced 624.Mel 38 (HLA-A2^+^), SK.Mel 37 (HLA-A2^+^), LB373.Mel (HLA-A2^+^), BV173 (HLA-A2^+^) and MZ2.Mel 43 (HLA-A2^-^) cell lines. Expression was determined by GFP. **(C)** IFNγ production of T9141- or T8922-transduced human T cells after co-culturing with ABL-minigene-E255V-transduced or untransduced cancer cell lines, as well as cancer cells loaded with 10^-6^ M ABL-E255V or ABL-E255WT. Unspecific T cell stimulation with PMA/ionomycin (P/I) was used as positive control. Mean values of duplicate cultures with SD are shown. Two-way ANOVA followed by Tukey’s multiple comparisons test was performed for statistical analysis (*P < 0.05, ****P < 0.0001; ns, not significant). Data are representative of 3 independent experiments of different donors. **(D)** Expression of HLA-A2 of tested cancer cell lines. Expression was determined by HLA-A2 staining.

To verify the expression of ABL-E255V in human cells and the recognition by specific TCRs, we co-cultured TCR-transduced T cells with cancer cells transduced with ABL-minigene-E255V. This minigene is an approximately 1 kb DNA fragment derived from *ABL* gene exon 1 to exon 6 with the point mutation E255V in exon 4 (**Figure 3A**). As determined by GFP expression, the three transduced melanoma cell lines, 624.Mel 38, SK.Mel 37 and LB373.Mel, expressed comparable levels of ABL-minigene-E255V, whereas the transduced CML cell line, BV173, had a lower level of expression (**Figure 3B**). The HLA-A2^-^ melanoma cell line, MZ2.Mel 43, was included as control.

Both isolated TCRs recognized HLA-A2^+^/ABL-minigene-E255V^+^ cells (**Figure 3C**). T9141-TCR exhibited significant responses, despite a weaker response to transduced BV173 cells. This result was consistent with the expression level of minigene detected in the transduced cell lines. In contrast, T8922-TCR showed a robust response to transduced 624.Mel 38 cells, but exhibited significantly reduced responses to transduced SK.Mel 37 and LB373.Mel cells, and unexpectedly failed to recognize transduced BV173 cells, suggesting that T8922-TCR has a too low avidity. On the other hand, these results showed that the transduced cell lines expressed different levels of ABL-E255V despite comparable levels of minigene and HLA-A2 expression, indicating that the efficiency of peptide processing varies among cell lines (**Figures 3B, D**). In summary, ABL-E255V is processed and presented in human cells and is recognized by ABL-E255V-specific TCRs.

### T9141-TCR exhibit no allo- and cross-reactivity

**Figure 4:**
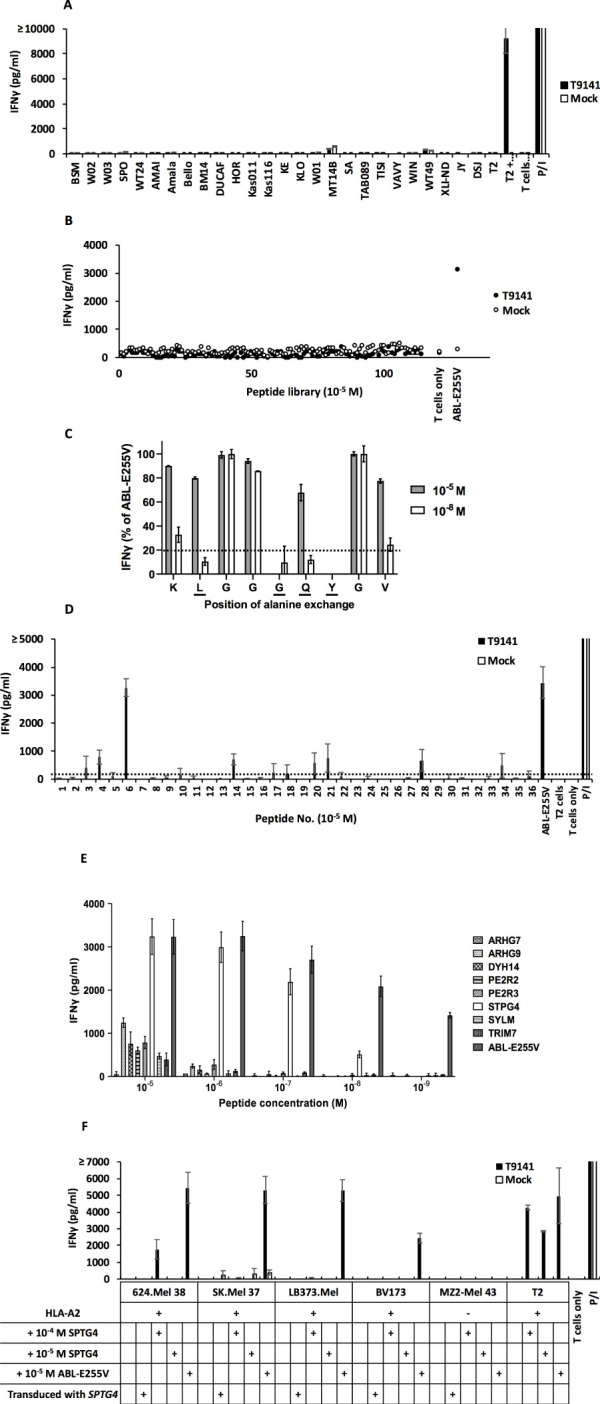
AB*ab*-A2 mouse-derived T9141-TCR exhibits no allo-reactivity and no cross-reactivity. **(A)** IFNγ production of T9141-transduced human T cells after co-culturing with a panel of B-LCLs expressing variable HLA allotypes. Unspecific T cell stimulation with P/I was used as positive control. Mean values of duplicate cultures with SD are shown. Data are representative of 2 independent experiments of 2 donors. **(B)** IFNγ production of T9141-transduced human T cells after co-culturing with T2 cells loaded with 114 different HLA-A2-restricted human self-peptides or ABL-E255V at 10^-5^ M. T2 cells without peptide loading (W/O) was used as negative control. Data are representative of 2 donors. **(C)** IFNγ production of T9141-transduced human T cells after co-culturing with T2 cells loaded with 9 different peptides at either 10^-5^ or 10^-8^ M. Each peptide contains one alanine substitution sequentially from ABL-E255V. Data were normalized to IFNγ production in response to ABL-E255V peptide. Dash line indicates a threshold of 20% of IFNγ production. Underlined amino acids indicate T9141 recognition motif. Mean values of duplicate cultures with SD are shown. Data are representative of 3 independent experiments of different donors. **(D)** IFNγ production of T9141-transduced human PBMCs after co-culturing with T2 cells loaded with 36 peptides containing the T9141 recognition motif (x-L-x-x-G-Q-Y-x-x) at 10^-5^ M. T cell stimulation with ABL-E255V and P/I were used as positive control, respectively. The 36 peptides carrying the T9141 recognition motif are listed in **Supplementary Table S6**. Dash line indicates a threshold of 250 pg/ml of IFNγ production. The diagram shows mean values of 2 independent experiments of 2 donors with SD. **(E)** IFNγ production of T9141-transduced human PBMCs after co-culturing with T2 cells loaded with 8 different peptides with down-titrated peptide concentrations. The diagram shows mean values of 2 independent experiments with SD. **(F)** IFNγ production of T9141-transduced T cells after co-culturing with *STPG4*-transduced, untransduced cancer cell lines and cells loaded with STPG4 or ABL-E255V peptide at indicated concentrations. Unspecific T cell stimulation with P/I was used as positive control. Mean values of duplicate cultures with SD are shown. Data are representative of 3 independent experiments of different donors.

To prevent unforeseen immune responses, T9141-TCR was tested for off-target toxicity. As AB*ab*-A2 mice express HLA-A2 but no other human HLA alleles, we analyzed allo-reactivity of T9141-TCR by co-culturing TCR-transduced T cells with a panel of B lymphoblastoid cell lines (B-LCLs) expressing different HLA molecules (**Supplementary Table S4**). No allo-reactivity was detected (**Figure 4A**).

As T9141-TCR was selected in the murine thymus, it may respond non-specifically to certain human peptides. First, we analyzed the cross-reactivity of T9141-TCR with 114 known HLA-A2-restricted human self-peptides loaded on T2 cells at 10^-5^ M (32). No cross-reactivity was detected (**Figure 4B**). Next, we analyzed the recognition motif of T9141-TCR by measuring its response to ABL-E255V mutated sequentially to alanine at each position (**Supplementary Table S5**). Position 2, 5, 6, and 7 (x-L-x-x-G-Q-Y-x-x) were identified as the TCR binding motif (**Figure 4C**). Of 36 human peptides containing this motif but which were absent in mice (**Supplementary Table S6**), eight peptides were evaluated as candidates for cross-reactivity (**Figure 4D**). Here, peptides eliciting responses below 250 pg/ml of IFNγ release were neglected, because peptides that are poorly recognized at 10^-5^ M are unlikely to elicit responses at physiological levels. The 8 peptides were investigated by titration assay, of which one peptide derived from the *STPG4* gene (sperm-tail PG-rich repeat containing 4) was recognized by T9141-TCR at a concentration as low as 10^-8^ M (**Figure 4E**). We further co-cultured T9141-transduced T cells with full-length *STPG4*-transduced cancer cells (**Supplementary Figures S1A, B**). No recognition was detected (**Figure 4F**). Importantly, T9141-transduced T cells responded poorly to all cell lines externally loaded with STPG4, except T2 cells, even at 10^-4^ M, suggesting that large amounts of STPG4 are needed to compete with endogenous peptides for HLA-A2. In other words, STPG4 peptide is unlikely to cause cross-reactivity, as it cannot be expressed at such high levels under normal physiological conditions. Taken together, no relevant cross-reactivity of T9141-TCR was detected.

### T cells expressing T9141-TCR recognize one of the two mutated CML cell lines harboring the E255V mutation

**Figure 5:**
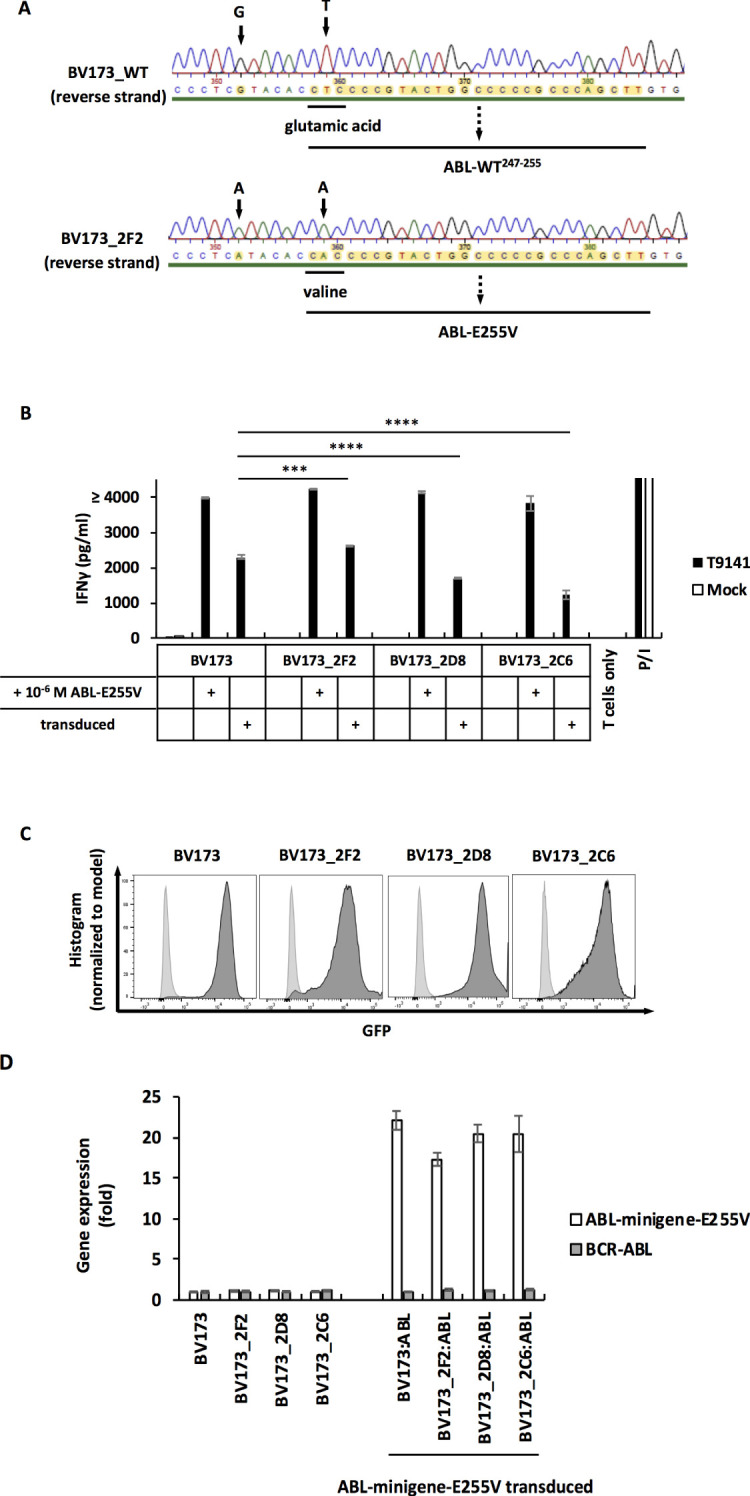
The mutated BV173 clones are not recognized by T9141-transduced T cells despite functional processing machinery and presentation of peptides. **(A)** Sequencing of a specified region on the *BCR-ABL* gene that was amplified from cDNA. Results of reverse strand are shown. Upper result is the sequencing of unmodified BV173 cells (BV173_WT); lower result is the sequencing of the mutated clone, BV173_2F2, which is representative of 3 mutated clones. BV173 cells were CRISPR-Cas9-edited by using crRNA3 and ssODN3. **(B)** IFNγ production of T9141-transduced human T cells after co-culturing with untransduced and ABL-minigene-E255V-transduced mutated BV173 clones, and cells loaded with 10^-6^ M ABL-E255V. Unspecific T cell stimulation with P/I was used as positive control. Mean values of duplicate cultures with SD are shown. Two-way ANOVA followed by Tukey’s multiple comparisons test was performed for statistical analysis (***P < 0.001, ****P < 0.0001). The results are representative of 2 independent experiments of 2 donors. **(C)** Expression of ABL-minigene-E255V in transduced BV173 cells and three mutated BV173 clones. Expression was determined by GFP. **(D)** Gene expression by RT-PCR of the *BCR-ABL* gene and ABL-minigene-E255V in parental BV173 cells and three mutated BV173 clones that were either untransduced or ABL-minigene-E255V-transduced. The value of gene expression is presented as relative quantification (RQ) in fold calculated by the method 2^-(ΔΔ^ *^Ct^* ^)^ ± RQ_max_ and RQ_min_.

The processing and presentation of ABL-E255V has been demonstrated by ABL-minigene-E255V-transduced cancer cells (**Figure 3**). However, it was important to investigate mutated CML cells that acquire the E255V mutation in the endogenous *BCR-ABL* gene, as the overexpressed minigene may not accurately reflect the natural scenario. Due to the lack of CML cell lines that are HLA-A2^+^/E255V^+^, we performed CRISPR-Cas9 to introduce the E255V mutation into BV173 cells. After screening and sequencing (**Supplementary Figure S2**), three mutated BV173 clones were obtained (**Figure 5A**). We then co-cultured T9141-transduced T cells with the three mutated BV173 clones. Unexpectedly, T cell responses were elicited only when the mutated BV173 clones were externally loaded with ABL-E255V peptide (**Figure 5B**). To assess the functionality of the clones’ peptide processing machinery, the mutated BV173 clones were transduced with ABL-minigene-E255V for co-culture (**Figures 5B, C**). Although all minigene-transduced cell samples elicited specific responses, the mutated BV173 clones on average induced lower IFNγ production than the parental cells, suggesting that the mutated BV173 clones had a less efficient processing machinery after a series of manipulations and long-term culture. As BCR-ABL is required for neoplastic transformation and survival of leukemic cells in CML, its overexpression is frequently observed (33–36). To compare the expression of the *BCR-ABL* gene and ABL-minigene-E255V in each BV173 sample, we performed q-PCR. In contrast to the *BCR-ABL* gene, the expression of ABL-minigene-E255V was 20-fold higher (**Figure 5D**). These results may explain why the mutated BV173 clones were not recognized, since they expressed the *BCR-ABL* gene at much lower levels than the minigene and had a less efficient processing machinery, thereby presenting insufficient levels of ABL-E255V for T cell recognition.

**Figure 6:**
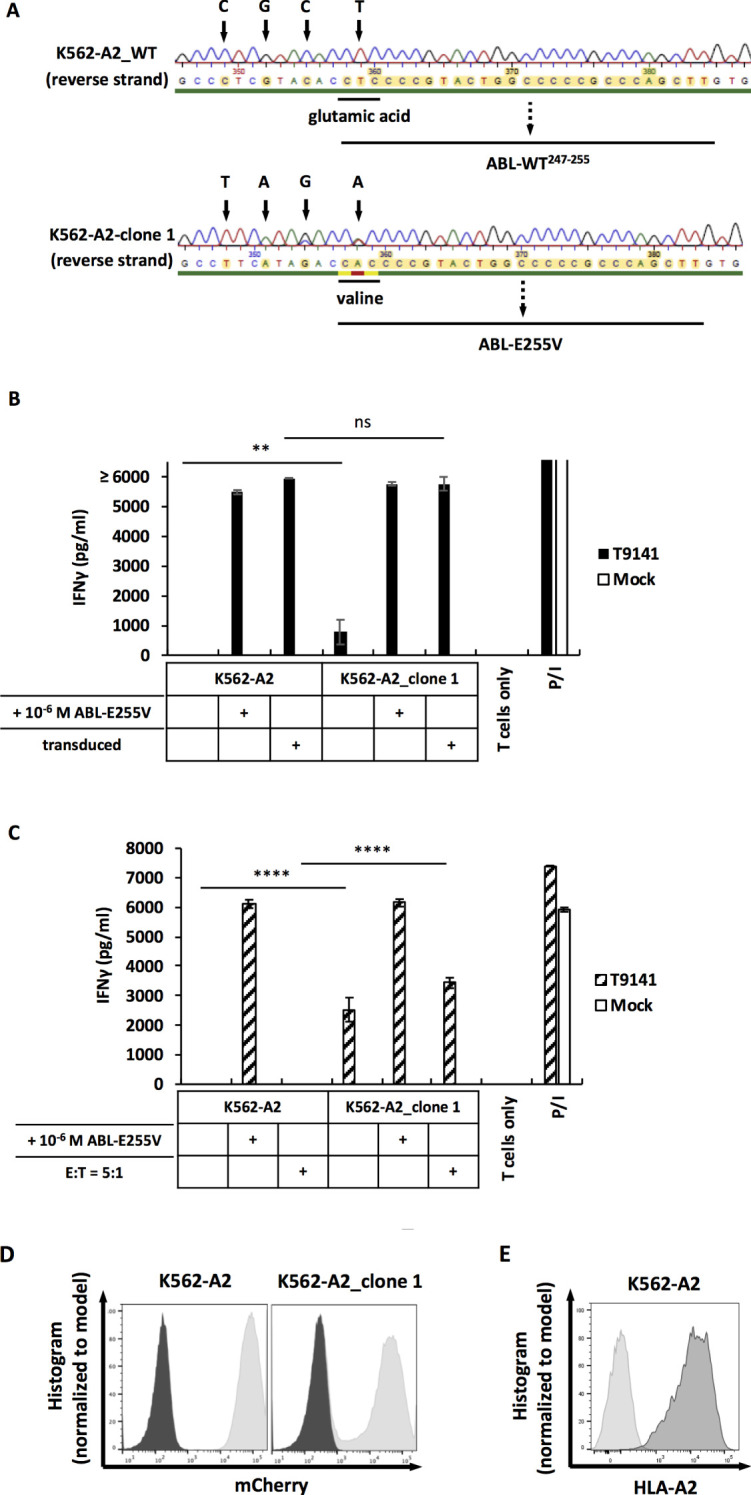
The mutated K562-A2 clone can be recognized by T9141-transduced T cells. **(A)** Sequencing of a specified region on the *BCR-ABL* gene that was amplified from cDNA. Results of reverse strand are shown. Upper result is the sequencing of unmodified K562-A2 cells (K562-A2_WT); lower result is the sequencing of the mutated K562-A2_clone 1. K562-A2 cells were CRISPR-Cas9-edited by using crRNA6 and ssODN6. **(B)** IFNγ production of T9141-transduced human T cells after co-culturing with untransduced and ABL-minigene-E255V-transduced K562-A2_clone 1 (E:T=1:1), as well as cells loaded with ABL-E255V at 10^-6^ M. Unspecific T cell stimulation with P/I was used as positive control. Mean values of duplicate cultures with SD are shown. The results are representative of 2 donors. **(C)** IFNγ production of 5×10^4^ T9141-transduced human T cells after co-culturing with K562-A2_clone 1 at E:T ratio of 1:1, as well as cells loaded with ABL-E255V at 10^-6^ M. Co-cultures with an E:T ratio of 5:1 were separately indicated. Unspecific T cell stimulation with P/I was used as positive control. Mean values of triplicate cultures with SD are shown. Data are representative of 2 independent experiments of 2 donors. **(D)** Expression of ABL-minigene-E255V in transduced K562-A2 and K562-A2_clone 1 cells. Expression was determined by mCherry. **(E)** Expression of HLA-A2 in transduced K562 (designated as K562-A2). Expression was determined by HLA-A2 staining. **(B, C)** Two-way ANOVA followed by Tukey’s multiple comparisons test was performed for statistical analysis (**P < 0.01, ****P < 0.0001; ns, not significant).

As CML cell lines can express varying levels of BCR-ABL and have different processing efficiencies, we included an additional CML cell line, K562, transduced to express HLA-A2 and edited using CRISPR-Cas9. Through screening and sequencing, the K562-A2 clone 1 was determined to carry the E255V mutation in the *BCR-ABL* gene (**Figure 6A**). After co-cultured with T9141-transduced T cells, this mutated clone induced a specific, albeit weak T cell response (**Figure 6B**). Nonetheless, T cell response to the mutated K562-A2 clone was significantly improved by using a higher number of transduced T cells and by adjusting the ratio of effector cells to target cells to 5:1 (**Figure 6C**). These results demonstrate that ABL-E225V derived from the mutated *BCR-ABL* gene can be presented in HLA-A2^+^ CML cells. Additionally, the mutated K562-A2 clone that underwent CRISPR-Cas9-editing and screening exhibited a well-functional peptide processing machinery, as the mutated clone transduced with ABL-minigene-E255V elicited a T cell response comparable to transduced parental cells (**Figures 6B, D**). And K562-A2 cells were also verified to express similar levels of HLA-A2 as cell lines that naturally express HLA-A2 (**Figures 3D**, **6E**). In summary, T9141-transduced T cells can recognize HLA-A2^+^/E255V^+^ CML cells. Although the response is highly dependent on the BCR-ABL expression in CML cells, it can be improved by optimizing the number of TCR-transduced T cells.

## Discussion

Relapse associated with secondary mutated BCR-ABL remains a problem in CML, despite different generations of TKIs. Importantly, compound mutations arising after sequential TKI treatment have been shown to confer differential resistance to most available TKIs (13, 14). Nevertheless, secondary mutations can be ideal targets for adoptive T cell therapy due to their cancer-specificity. In this study, we immunized AB*ab*-A2 mice expressing a diverse human TCR repertoire restricted to HLA-A2 with mutated peptides to test corresponding T cell responses, characterized TCRs isolated from immune-responding mice for clinical use, and examined the processing of the target mutated peptide in human cancer cells.

In Europe, the annual number of HLA-A2^+^/E255V^+^ CML patients is estimated to be between 14 to 34, while the annual number of HLA-A2^+^/T315I^+^ CML patients is estimated to be up to 110 (37–41). Although the cases of E255V is relatively low, E255V is significantly associated with drug-resistance. Here, we demonstrated that ABL-E255V is a naturally processed and presented neoepitope. Secondary mutations in BCR-ABL have been studied as newly arising targets for adoptive T cell therapy. A previous study by Cai et al. has shown that an E255K mutation-derived peptide, a good binder to HLA-A3, induced a specific T cell response in HLA-A3^+^ human PBMCs (42). In addition, they demonstrated the processing of the E255K-mutant peptide by detecting T cell reactivity to minigene-transfected antigen-presenting cells. However, it remains unclear whether the E255K mutant peptide is truly processed and presented in secondary mutated CML cells (43). In this study, we examined the expression of ABL-E255V in human cancer cells transduced with ABL-minigene-E255V, as well as mutated CML cells carrying the E255V mutation in the *BCR-ABL* gene. The recognition of transduced cancer cells demonstrates the processing and presentation of ABL-E255V. Importantly, we observed varying degrees of T cell responses against transduced cancer cell lines. The differences are likely due to varying amounts of ABL-E255V presented on the cell surface, which are influenced by the expression level of HLA-A2 and the minigene, as well as the antigen processing efficiency of each cell line. To further investigate the feasibility of targeting ABL-E255V, we tested T9141-transduced T cells with two mutated CML cell lines. The recognition of the mutated K562-A2 cell line demonstrates that ABL-E255V is truly processed from the endogenous BCR-ABL protein. Although the T cell response induced by mutated K562-A2 cells was weak compared to that induced by minigene-transduced K562-A2 cells, the T cell response could be significantly improved by adjusting the number of specific T cells.

Nevertheless, no recognition of the mutated BV173 cell line was observed which was surprising. Since neoplastic transformation in CML is highly dependent on the BCR-ABL protein (33–35), notable peptides derived from this protein are expected to be presented on the cell surface. Therefore, we hypothesize that inefficient peptide processing, resulting in inadequate ABL-E255V expression, may be responsible for the lack of recognition of mutated BV173 cells. We observed, despite having similar expression levels of ABL-minigene-E255V after transduction, that mutated BV173 cells elicited a lower T cell response compared to parental BV173 cells. This result suggests that mutated BV173 cells, through a series of manipulations and long-term culture, have decreased peptide processing efficiency. Accordingly, mutated BV173 cells may express ABL-E255V from the BCR-ABL protein at a level lower than it should be. Moreover, q-PCR analysis of transduced BV173 cells detected that the LTR-enhanced minigene expression was 20-fold higher than the *BCR-ABL* gene expression. Transduced parental BV173 cells elicited a lower T cell response among all transduced cancer cell lines in ABL-E255V processing assay (**Figure 3C**), suggesting that transduced parental BV173 cells, despite artificially enhanced minigene expression, have a relatively less efficient peptide processing compared to the other cell lines. Thus, the BCR-ABL protein naturally expressed in BV173 cells is expected to be processed and presented at an even lower level. Besides peptide processing efficiency, the BCR-ABL expression level should also be considered, as it directly affects the amount of ABL-E255V presented. A conspicuous level of BCR-ABL protein has been detected in BV173 cells. Nevertheless, the expression in BV173 cells was lower than in some CML cell lines such as K562 cells (44, 45), which renders BV173 cells less competent to elicit a specific T cell response. In fact, BCR-ABL expression varies widely among CML cell lines (44–47), highlighting the need to include different CML cell lines in the test.

The T9141-TCR derived from ABab-A2 mice is verified as a potential therapeutic TCR. By demonstrating that ABL-E255V is immunogenic and naturally processed, the isolation of specific TCRs from HLA-A2^+^/E255V^+^ CML patients may be possible. Nevertheless, chronic persistence of antigens has been demonstrated to cause exhaustion and deletion of high-avidity T cells (48–50), which has also been observed in CML (51, 52). Therefore, allogeneic donors and humanized transgenic mice become optimal sources for isolating TCRs. Here, T9141-TCR derived from ABab-A2 mice is verified as a therapeutic TCR, as no off-target toxicity was detected. Moreover, T9141-TCR exhibits a superior avidity (EC_50_ < 10^-10^ M). This avidity may still be insufficient, as T9141-TCR had no response or a low response to mutated BV173 and mutated K562-A2 cells, respectively. However, this avidity was only slightly weaker than that of T1367-TCR, a highly sensitive MAGE-A1 TCR (**Supplementary Figure S3**) (20), suggesting that T9141-TCR should be efficient. Theoretically, TCR-maturation *in vitro* can increase avidity, but the impact on the specificity and functionality of TCRs should be considered (32, 53, 54).

A limitation of this study is a lack of HLA-A2^+^/E255V^+^ CML cell lines. As different levels of BCR-ABL expression have been observed not only in CML cell lines but also in patients (55–57), the two mutated CML cell lines used in this study may inadequately represent the broader CML patient population. Importantly, upregulated BCR-ABL expression has been observed in accelerated and blast phase of CML (58–60), as well as under drug treatment (61), which may allow for strong responses mediated by T9141-transduced T cells. Therefore, including a broader range of CML cell lines in future studies should provide a comprehensive view of the efficacy of T1914-TCR in treating CML.

In summary, we identified the neoepitope ABL-E255V from secondary mutated CML and generated specific TCRs, in which the T9141-TCR was verified as a therapeutic candidate, as no off-target toxicity was detected. This study provides a new therapeutic option for CML patients resistant to TKIs and highlights the potential of adoptive T cell therapy in CML treatment, as new targets may be uncovered by screening secondary mutations in the BCR-ABL with a broad spectrum of HLA alleles.

## Data Availability

The original contributions presented in the study are included in the article/**Supplementary Files**, further inquiries can be directed to the corresponding author/s.
