# Supplementary material for: T cell receptors specific for an imatinib-induced mutation in BCR-ABL for adoptive T cell therapy
Source: Front Immunol. 2025 Jan 27;16:1518691. doi: 10.3389/fimmu.2025.1518691 (PMC11807957; doi:10.3389/fimmu.2025.1518691)
Supplement: Supplementary file 1 [file DataSheet1.pdf]

Supplemental data:

Supplemental Table 1. Description and maintenance of cell lines

Name	Description	Medium	HLA-A2
HEK-GALV	Human embryonic kidney cells expressing stably GALV-env and MLV-gag/pol	DMEM, 10% FCS (PAN Biotech)	-
T2	Human lymphoma cells defective in TAP	RPMI, 10% FCS	-
BV173	Human CML cells	RPMI, 20% FCS	+
K562	Human CML cells	RPMI, 10% FCS	-
LB373.Mel	Human melanoma cells	HMC medium: DMEM, 50 µM 2-mercaptoethanol, 1 mM sodium pyruvate and 1× nonessential amino acids	+
SK.Mel 37	Human melanoma cells	HMC medium	+
624.Mel 38	Human melanoma cells	HMC medium	+
MZ2.Mel 43	Human melanoma cells	HMC medium	-
LCLs	Lymphoblastoid cell lines	RPMI, 10% FCS, 50 µM 2-mercaptoethanol, 1 mM sodium pyruvate and 1× nonessential amino acids	*

* The HLA alleles of each LCL are listed in supplementary Table 4.

Supplemental Table 2. Single strand oligo DNA for CRISPR-Cas9-editing

Name	Sequence (5’ to 3’)*
ssODN3	TGGAACGCACGGACATCACCATGAAGCACAAGCTGGGCGGGGGCCAGTACGGGG**T**GGTGTA**T**GAGGGCGTGTGGAAGAAATACAG**T**CTGACGGTGGCCGTGAAGACCTTGAAGGTAGGCTGGGACTG
ssODN6	CCCGGCAGTCCCAGCCTACCTTCAAGGTCTTCACGGCCACCGTCAGGCTGTATTTCTTCCACACGCC**T**TC**A**TA**G**ACC**A**CCCCGTACTGGCCCCCGCCCAGCTTGTGCTTCATGGTGATGT

*Bold letters indicate exchanged nucleotides for amino acid mutations or silence mutations, where silence mutations are used to alter the recognition sequence of HPY8I enzyme to screen for mutated clones, or to alter the PAM sequence to disable sequential CRISPR-Cas9-editing in mutated clones. Underlined sequences indicate where the targeted glutamic acid is mutated to valine.

Supplemental Table 3. Primers for screening and sequencing of genomic edited clones

Primers	Sequence (5' to 3')
ABL-CRISPR-F	TGGCACCCACTGCATTGTTGCTTTC
ABL-CRISPR-R	TGCAGAATTGGAACCCACTGGGGATG
BCR_exon 9-F	AGCTGCTGAAGGACAGCTTCATGGTG
ABL_a7-R	TGGAGAACTTGTTGTAGGCCAGGCTC
ABL_a7_inner-R	GCCAAAATCAGCTACCTTCACC

Supplemental Table 4. HLA allotypes expressed by the B lymphoblastoid cell lines (B-LCLs). Part of B-LCLs are homozygous in all MHC-I loci. Allele designations follow the 2004 HLA nomenclature report.

B-LCL	A*		B*		Cw*	
AMAI	68:02		53:01		04:01	
AMAL	02:17:01		15:01:01:0		03:03	
Bello	02:02	11:01	41:01	52:01	12:02	17:01
BM14	03:01		07:02		07:02	
BSM	02:01:01		15:010101		03:04:01	
DSJ	02:01	03:01	35:01	37:02	04:01	06:02
DUCAF	30:02		18:01		05:01	
HOR	33:03:01		44:03:01		14:03	
JY	02:01		07:02:01		07:02:01:0	
KAS01	01:0101		37:01		06:02	
KAS11	24:020101		51:01		12:03	
KE	02:01	29:02	44:03	44:05	02:02	16:01
KLO	02:08	01:01:01:0	08:01:01	50:01:01	07:01:01:0	06:02:01:0
MT14	31:01		40:01		03:04	
SA	24:02:01:0		07:02:01		07:02	
SPO	02:01		44:02		05:01	
TAB08	02:07		46:01		01:02	
TISI	24:02:01:0		35:08		04:01	
VAVY	01:01		08:01		07:01	
W-01	03:01	24:02	15:01	35:01	03:03	04:01
W-02	02:01	26:01	38:01	44:02	38:01	44:02
W-03	02:01	23:01	15:01	58:01	03:04	07:01
WIN	01:01		57:01:01		06:02	
WT24	02:0101		27:0502		02:0202	
WT49	02:05:01		58:0101		07:18	
XLI-ND	02:10	30:01	13:02	40:06:01:0	06:02	08:01

Supplemental Table 5. Sequences of peptides used in the alanine scan for identifying the T9141-TCR recognition motif. The valine (V) point mutation in ABL-E255V peptide is underlined. The alanine (A) that replaces the original amino acid in each peptide is shown in bold.

Peptide	Sequence								
ABL-E255V	K	L	G	G	G	Q	Y	G	V
ABL-E255V-1A	**A**	L	G	G	G	Q	Y	G	V
ABL-E255V-2A	K	**A**	G	G	G	Q	Y	G	V
ABL-E255V-3A	K	L	**A**	G	G	Q	Y	G	V
ABL-E255V-4A	K	L	G	**A**	G	Q	Y	G	V
ABL-E255V-5A	K	L	G	G	**A**	Q	Y	G	V
ABL-E255V-6A	K	L	G	G	G	**A**	Y	G	V
ABL-E255V-7A	K	L	G	G	G	Q	**A**	G	V
ABL-E255V-8A	K	L	G	G	G	Q	Y	**A**	V
ABL-E255V-9A	K	L	G	G	G	Q	Y	G	**A**

Supplemental Table 6. Peptides carrying the T9141 recognition motif within the human proteome. Peptides in this table are not present in the mouse proteome. Predicted binding affinity of each peptide is analyzed by NetMHC 4.0. Peptides that elicit responses from T9141-transduced T cells at 10^{-5} M are shown in bold and have a gray background.

Peptide No.	Gene	Sequence with motif x-L-x-x-G-Q-Y-x-x	IC_{50} (nM)
1	ADIPL	QLQAGQYAS	9360
2	AGRF3	YLPQGQYLR	7360
**3**	**ARHG7**	**KLFQGQYRS**	**991**
**4**	**ARHG9**	**PLNHGQYLV**	**1474**
5	BAZ2B	KLSSGQYPN	9689
**6**	**CB061/STPG4**	**QLSPGQYNV**	**51**
7	CD69	ALSVGQYNC	5847
8	CF132	DLRPGQYGQ	35842
9	CGAT1	QLRNGQYQA	6984
10	CP8B1	KLDFGQYAK	11220
11	CR018	GLPPGQYAT	1937
12	CSPG4	ALKNGQYWV	98
13	DAPLE	PLKPGQYVK	39446
**14**	**DYH14**	**GLPHGQYSV**	**49**
15	ERFE	NLTSGQYRA	1563
16	ITB2	KLIYGQYCE	14103
17	LRP2	ILERGQYCK	28111
18	NU133	LLSLGQYLW	6817
19	OBSCN	TLREGQYVE	29891
**20**	**PE2R2**	**LLDYGQYVQ**	**13666**
**21**	**PE2R3**	**VLGVGQYTV**	**46**
22	PGBD1	LLERGQYPY	17251
23	PHF19	KLTEGQYVL	23
24	RRP5	KLKVGQYLN	27187
25	SARAF	FLSDGQYSP	527
26	SETD9	PLAVGQYVN	37417
27	SNX20	CLRAGQYPR	21812
**28**	**SYLM**	**RLPSGQYLQ**	**16208**
29	TBC31	ALTKGQYPV	14
30	TDRD3	QLHQGQYRS	18288
31	TENS3	KLSLGQYDN	25163
32	TM2D1	DLKVGQYIC	29048
33	TRI69	KLNLGQYKG	15168
**34**	**TRIM7**	**QLNGGQYWA**	**480**
35	ZAN	QLKNGQYGC	20705
36	ZMIZ2	YLQGGQYAP	2867

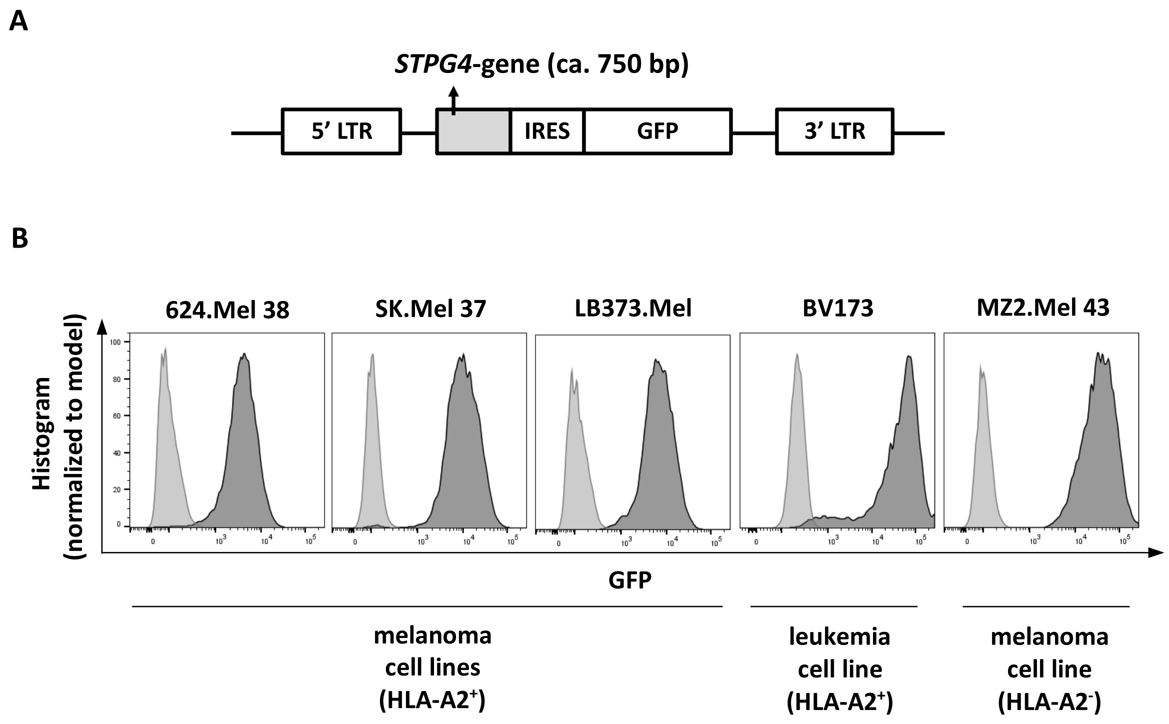

Supplemental Figure 1. Human cancer cell lines are transduced with a full length of *STPG4* gene. (A) Schematic of the *STPG4* gene linked to a GFP reporter gene by IRES and flanked with 5' and 3' LTR in pMP71 vector. (B) Expression of *STPG4* in transduced 624.Mel 38 (HLA-A2$^+$), SK.Mel 37 (HLA-A2$^+$), LB373.Mel (HLA-A2$^+$), BV173 (HLA-A2$^+$) and MZ2.Mel 43 (HLA-A2$^-$). Expression was determined by measuring GFP.

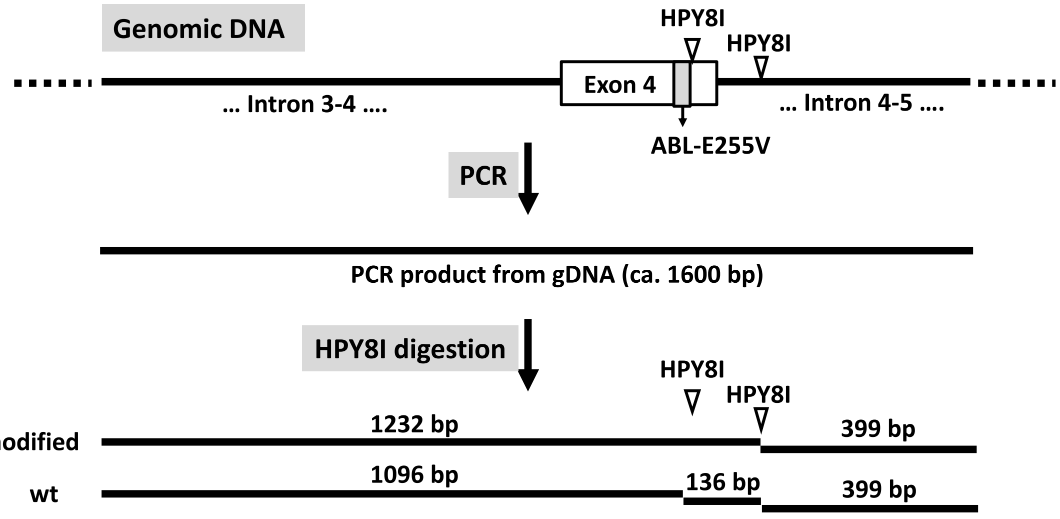

Supplemental Figure 2. The screening strategy for identifying CRISPR-edited cell clones. Schematic of screening strategy for identifying CRISPR-edited cell clones. PCR product was amplified from genomic DNA of each cell clone. Following digestion of HPY8I restriction enzyme, DNA fragment with a length of ca. 1.2 kb served as an indicator of clones carrying modified sequences.

A

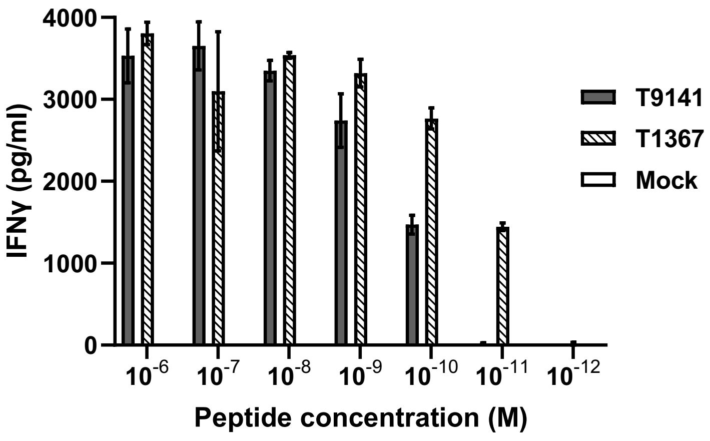

B

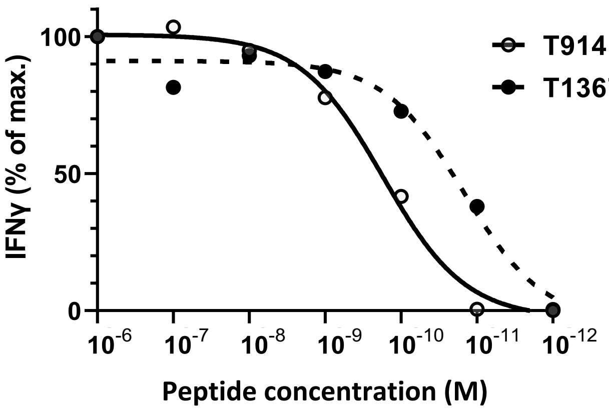

Supplemental Figure 3. T9141 TCR exhibited one-log lower functional avidity compared to MAGE-A1-specific T1367 TCR. (A) IFNγ production of T9141- and T1367-transduced human T cells after co-culturing with T2 cells loaded with ABL-E255V or MAGE-A1 peptide at 10^{-6} M to 10^{-12} M. Mean values of duplicate cultures with SD are shown. Data are representative of 3 independent experiments of different donors. (B) Responses of transduced human PBMCs were normalized to maximum IFNγ release.
